# Gprc5a-knockout mouse lung epithelial cells predicts ceruloplasmin, lipocalin 2 and periostin as potential biomarkers at early stages of lung tumorigenesis

**DOI:** 10.18632/oncotarget.14589

**Published:** 2017-01-10

**Authors:** Beibei Sun, Wenzheng Guo, Song Hu, Feng Yao, Keke Yu, Jie Xing, Ronghua Wang, Hongyong Song, Yueling Liao, Tong Wang, Pengfei Jiang, Baohui Han, Jiong Deng

**Affiliations:** ^1^ Translation Medicine Center, Shanghai Chest Hospital, Shanghai Jiao Tong University, Shanghai, China; ^2^ Key Laboratory of Cell Differentiation and Apoptosis of Chinese Minister of Education, Department of Pathophysiology, Shanghai Jiao Tong University School of Medicine, Shanghai, China; ^3^ Shanghai Key Laboratory for Tumor Microenvironment and Inflammation, Shanghai Jiao Tong University School of Medicine, Shanghai, China; ^4^ Department of Thoracic Surgery, Shanghai Chest Hospital, Shanghai Jiao Tong University, Shanghai, China; ^5^ Department of Biobank, Shanghai Chest Hospital, Shanghai Jiao Tong University, Shanghai, China; ^6^ Department of Laboratory Medicine, East Hospital, Tongji University School of Medicine, Shanghai, China; ^7^ Department of Pulmonary Medicine, Shanghai Chest Hospital, Shanghai Jiao Tong University, Shanghai, China

**Keywords:** lung cancer, biomarker, ceruloplasmin, Gprc5a

## Abstract

Lung cancer is the leading cause of cancer death. As most of lung cancer patients were diagnosed with the advanced stage, early detection is considered as the most effective strategy to reduce high mortality. Thus, it is desirable to identify specific biomarkers at early stages of lung tumorigenesis. GPRC5A is a lung tumor suppressor gene. GPRC5A deficiency is linked to lung cancer development. We hyposthesized that, dysregulated gene expression that results from Gprc5a deficiency may provide potential biomarkers at early stages of lung tumorigenesis. By analysis of top 20 upregulated genes in mouse tracheal epithelial cells (MTEC) of Gprc5a knockout (KO) vs wild-type (WT), we found that ceruloplasmin, lipocalin-2, and periostin are not only upregulated in lung epithelial cells of Gprc5a-ko mice, but also expressed at high levels in lung tumor tissues of Gprc5a-ko mice. This suggests that increased expression of these genes is associated with lung tumorigenesis. Importantly, expression of ceruloplasmin, lipocalin-2, and periostin has also been found to be significantly increased, both at mRNA and protein levels, in the lung tissues from NSCLC patients, which is correlated with repressed GPRC5A. Thus, dysregulated ceruloplasmin, lipocalin-2, and periostin may be used as potential biomarkers at early stages of lung tumorigenesis.

## INTRODUCTION

Lung cancer is the leading cause of cancer death worldwide, which account for approximately one third of all cancer mortality [1]. Despite continuous improvement in diagnosis and treatment of lung cancer, the 5-year survival rate for non-small cell lung cancer (NSCLC) remains low, approximately 15%. Clinically, over 70% of lung cancer patients are diagnosed with advanced stage, therefore early detection is crucial for reducing the mortality. It would be of great help for timely clinical intervention if specific and sensitive biomarkers that highlight the pathological changes at early stages of lung tumor development could be identified.

Tumor development is a multiple stage process, in which both activation of oncogenes and inactivation of tumor suppressor genes are involved. Up to now, over half of NSCLC are found with known driver oncogene mutations, the gain-of-function somatic mutations including EGFR, KRAS, BRAF, NRAS, PI3KCA, AKT, etc., whereas other half are without known driver oncogene mutations [2]. Emerging evidences have shown that, for solid tumor development, there are more genetic alterations of inactivation of tumor suppressor genes, such as genetic deletion, than those of activation of oncogenes [3]. Another point is that, in the progression of premalignant lesions to carcinoma of lung, there are cumulative gene losses involved. Thus, characterization of molecular changes driven by tumor suppressor gene deletion in the process of carcinogenesis may lead to identify biomarkers that occurred at early stages of lung tumor development.

Gprc5a, a lung tumor suppressor gene, is preferentially expressed in lung tissue [4, 5]. Gprc5a gene knockout (ko) leads to development of both spontaneous and carcinogen-induced lung cancer in mice [5, 6], indicating that Gprc5a deficiency is sufficient to initiate lung tumorigenesis. Importantly, repression of GPRC5A has been found in most of human lung cancer including lung adenocarcinomas (ADC) and squamouse cell carcinomas (SCC) [7]. Moreover, frequent loss of heterozygosity (LOH) of chromosome 12p13, where GPRC5A gene locus is located, was identified in approximately one third of NSCLC [8]. Mechanistically, Gprc5a gene depletion leads to aberrantly activated NF-κB and EGFR-STAT3 in mouse lung epithelial cells [6, 9], which is consistent with dysregulated signaling pathways in NSCLC. As Gprc5a deficiency leads to development of lung tumor, we hypothesized that, some of the molecular alterations that are resulted from Gprc5a deficiency might be associated with lung tumorigenesis, and may therefore be used as potential biomarkers for diagnosis at early stages of lung tumorigenesis.

In this study, we screened for upregulated genes in Gprc5a-ko vs wild-type (WT) mouse tracheal epithelial cells (MTEC) via expression array analysis. Among top 20 upregulated genes in KO-MTEC vs WT-MTEC, we selected ceruloplasmin (Cp), lipocalin-2 (LCN2), and periostin (Postn) as candidate genes for further investigation. The reasons to choose to these genes are: i) data from public domain ONCOMINE shows that Cp, LCN2 and Postn are upregulated in lung cancer verse normal lung tissues; ii) at least in some reports, these genes are upregulated in certain types of tumor samples; iii) the products of these genes can be detected in serum or liquid biopsy [10–12], which is applicable in early diagnosis. By immunoblot, Q-PCR, and immunohistochemical (IHC) staining, we found that ceruloplasmin, lipocalin-2, and periostin are not only upregulated in the lung tumor tissues of Gprc5a-ko mice, but also in human non-small cell lung cancer (NSCLC). Taken together, GPRC5A deficiency signature predicts ceruloplasmin, lipocalin-2, and periostin as potential biomarkers at early stages of lung tumorigenesis.

## RESULTS

### Gprc5a gene knockout in mouse tracheal epithelial cells predicts ceruloplasmin, lipocalin 2 and periostin among 20 most upregulated genes

**Figure 1:**
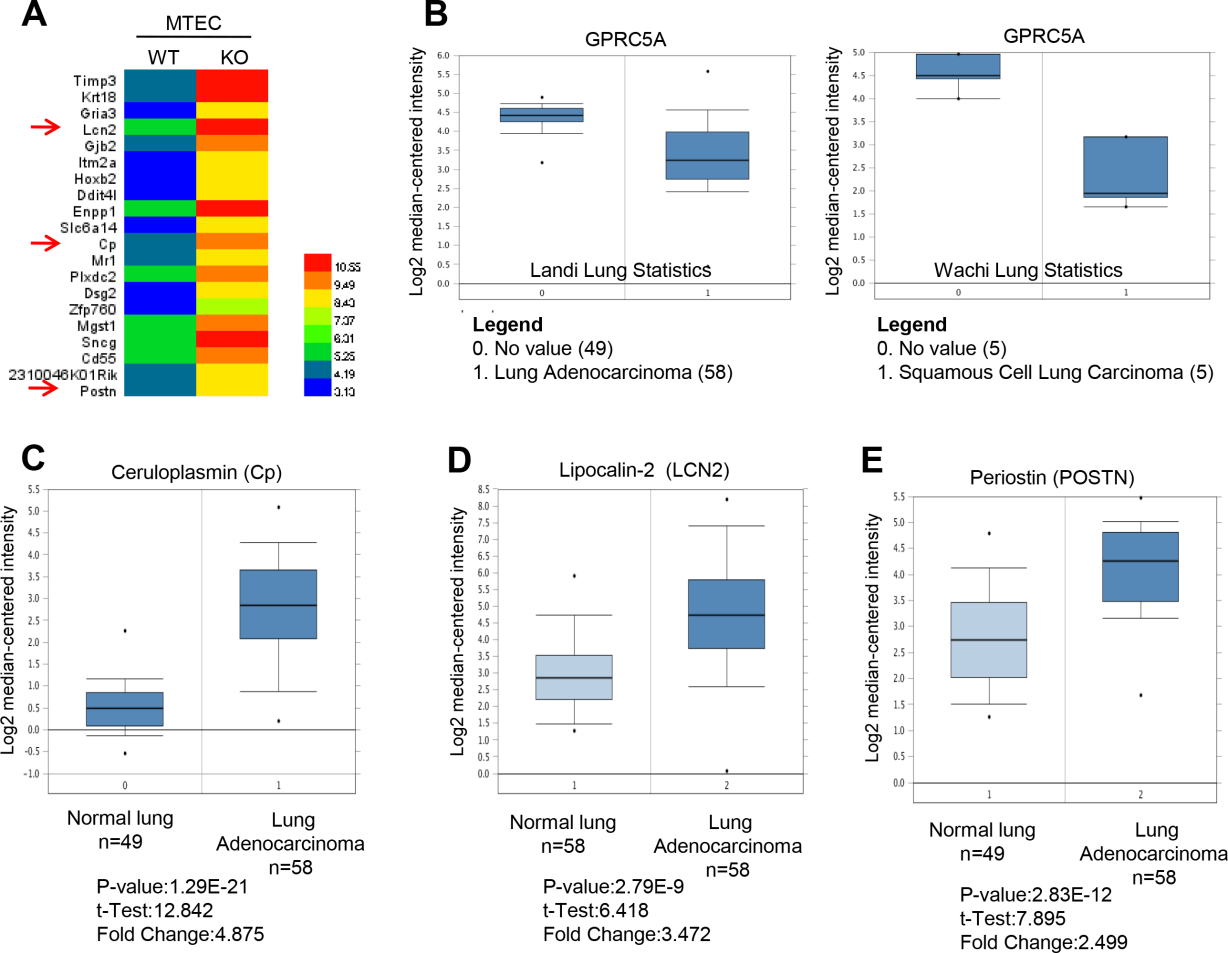
Ceruloplasmin, lipocalin 2 and perioston, among top 20 upregulated genes in Gprc5a knockout vs wild-type mouse tracheal epithelial cells are dysregulated in NSCLC based on data from ONCOMINE (**A**) Heat map of top 20 upregulated gene via expression array analysis in mouse tracheal epithelial cells (MTEC) of Gprc5a-knockout (KO) and wild-type (WT). (**B**) Relative mRNA expression levels of GPRC5A in human lung adencarcinoma (left) and squamous cell lung carcinoma (right) vs normal lung tissues. Relative expression of mRNA Ceruloplasmin (**C**), lipocalin 2 (LCN2) (**D**) and periostin (POSTN) (**E**) in human lung adenocarcinoma vs normal lung tissues based on data from ONCOMINE.

Gprc5a gene depletion leads to spontaneous lung tumor development in mouse model [5, 9], suggesting GPRC5A deficiency can initiate lung tumorigenesis. To screen for potential biomarkers at early stages of lung tumorigenesis that is initiated by Gprc5a deficiency, we first examined the gene expression pattern of Gprc5a-knockout (ko) vs wild-type (WT) tracheal epithelial cells (MTEC) via expression array. We then screened for potential candidate biomarker genes for further analysis among top 20 mostly upregulated genes in KO-MTEC compared to WT-MTEC as (Figure 1A, Supplementalry Table 1). We searched for the information on public domain in ONCOMINE and compared the expression pattern of these genes in lung cancer tissues vs normal tissues. While GPRC5A was downregulated in both human lung adenocarcinoma (Figure 1B, left) and squamous cell lung carcinoma (Figure 1B, right), several candidate genes were also found to be upregulated in lung cancer tissues compared to normal ones, including keratin 18 (Krt18), lipocalin 2 (LCN2), ceruloplasmin (Cp), major histocompatibility complex class I-related (Mr1), desmoglein 2 (Dsg2), ectonucleotide pyrophophatase/phosphodiesterase 1 (Enpp1), and periostin (Postn) (Figure 1C–1E).

**Table 1:** The baseline characteristics of NSCLC patients include in the study

Characteristics	Patients	
	NO.	%
Age, years		
< 60	39	41.1
≥ 60	56	58.9
Gender		
Male	74	77.9
Female	21	22.1
Pathological type		
ADC	59	62.1
SCC	36	37.9
T-primary tumor size		
T1	25	26.3
T2	61	64.2
T1 + T2	86	90.5
T3	7	7.4
T4	2	2.1
T3 + T4	9	9.5
N-regional lymph node		
Negative	60	63.2
Positive	35	36.8
TNM stage		
I	51	53.7
II	19	20.0
I + II	70	73.7
III	23	24.2
IV	2	2.1
III + IV	25	26.3
Histopathological type		
Grade 1–2	29	30.5
Grade 3	66	69.5
Smoking history		
Yes	25	26.3
No	68	71.6
NA*	2	2.1
Total	95	100

*Note: NA = not available (Two patients were lost to follow-up.)

**Figure 2:**
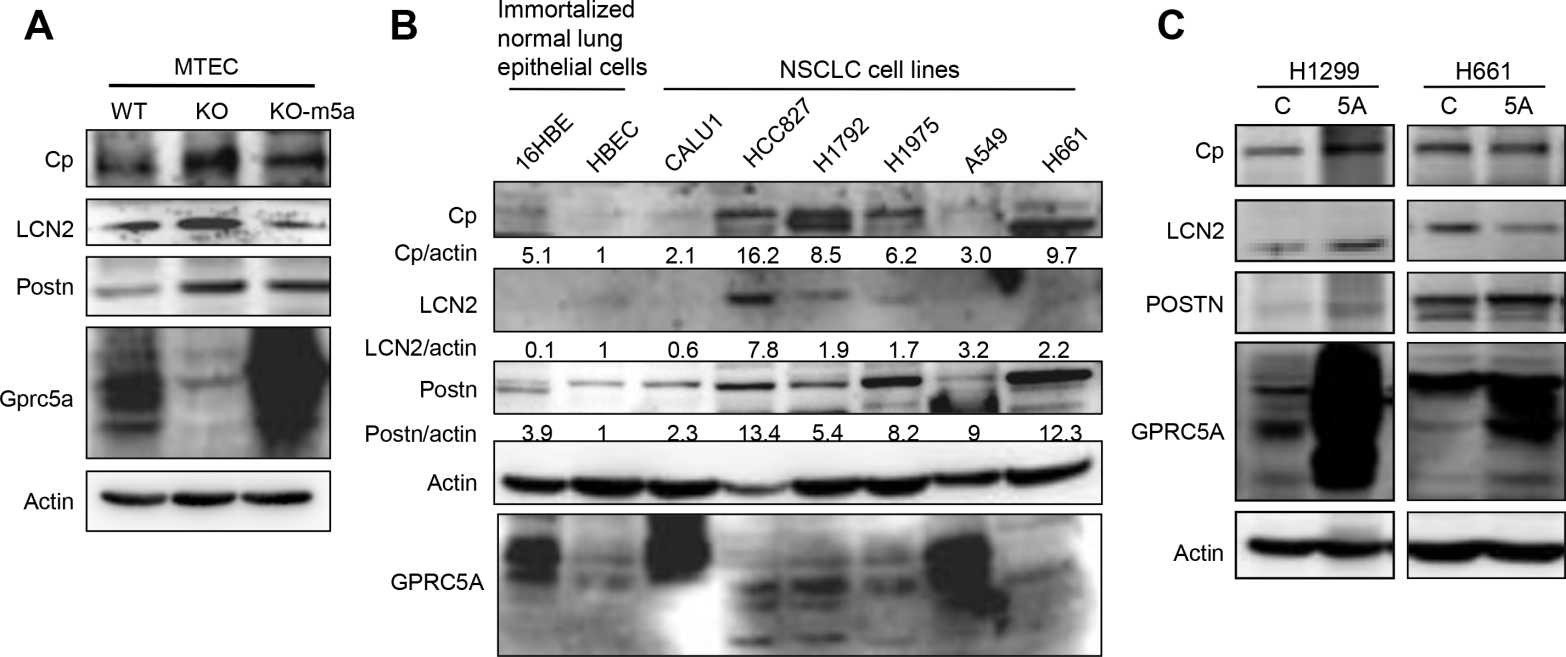
Protein levels of ceruloplasmin, lipocalin 2 and periostin in MTEC and NSCLC cell lines are correlated with repressed GPRC5A (**A**) Cell lysates from WT-, KO-MTEC, and Gprc5a transfectants of KO-MTEC (KO-m5a) were harvested and subjected to immunoblot analysis, with indicated antibodies to Cp, LCN2, Postn and Gprc5a. (**B**) Immunoblot analysis of cell lysates from six NSCLC cell lines (Calu1, HCC827, H1792, H1975, A549 and H661) and two normal human epithelial cell lines (16HBE, HBEC) with indicated antibodies were performed, relative expression levels are expressed as the ratio below the bands that are the intensities of indicated proteins verse actin. (**C**) Immunoblot of cell lysates from GPRC5A transfectants of NSCLC cells (5A in H1299 and H661).

Through literature search, we found that at least in some types of cancer, Cp, LCN2, and Postn have been reported to be upregulated. Thus, we performed further characterization on the regulation of these genes. By immunoblot analysis, we found that Cp, Postn and LCN2 were indeed significantly upregulated in KO-MTEC compared to WT-MTEC (Figure 2A). Importantly, re-expression of mouse Gprc5a in KO-MTEC (MTEC-KO-m5a) reduced the levels of Cp, LCN2, and, to less extend, Postn (Figure 2A). This suggests that, at least in normal mouse lung epithelial cells, Gprc5a expression can regulate the expression of these genes. Next, we examined the expression pattern of these genes in human NSCLC cell lines. The results of immunoblot showed that, the expression of Cp, LCN2 and POSTN is relatively low in immortalized human bronchiole epithelial cells, HBEC and 16HBE cells. However, the expression levels of Cp, LCN2, and Postn are relatively high in most of lung cancer cell lines including HCC827, H1792, H1975, and H661, but not in Calu-1 and A549. Interestingly, expression of GPRC5A in A549 and Calu-1 is relatively high (long exposure), whereas it is low in HCC827, H1792, H1975, and H661 (Figure 2B). It appears that GPRC5A expression is inversely correlated with the expression of these genes. To determine the effects of GPRC5A over-expression, we established stable GPRC5A transfectants of H1299 and H661 that have low level of GPRC5A. The results showed that the levels of Cp, LCN2, and Postn in NSCLC cells were not changed in H1299-GPRC5A (5A) and H661-5A in comparison with those of parent H1299 and H661 (Figure 2C). Thus, over-expression of GPRC5A is not sufficient to repress the upregulated Cp, LCN2, and POSTN in NSCLC cells. Presumably, the epigenetic changes during tumor development and progression prevent the inhibitory effects of GPRC5A overexpression on Cp, LCN2 and POSTN in NSCLC cells. Nevertheless, upregulated Cp, LCN2, and POSTN are identified both in Gprc5a-ko mouse lung epithelial cells and NSCLC cells, suggesting that upregulated expression of Cp, LCN2, and POSTN that results from GPRC5A deficiency may occur in normal lung epithelial cells or at an early stage, which can be associated with the process of lung tumorigenesis.

### High expression levels of Cp, LCN2 and Postn are maintained in lung tumor tissues in Gprc5a-ko mice *in vivo*

**Figure 3:**
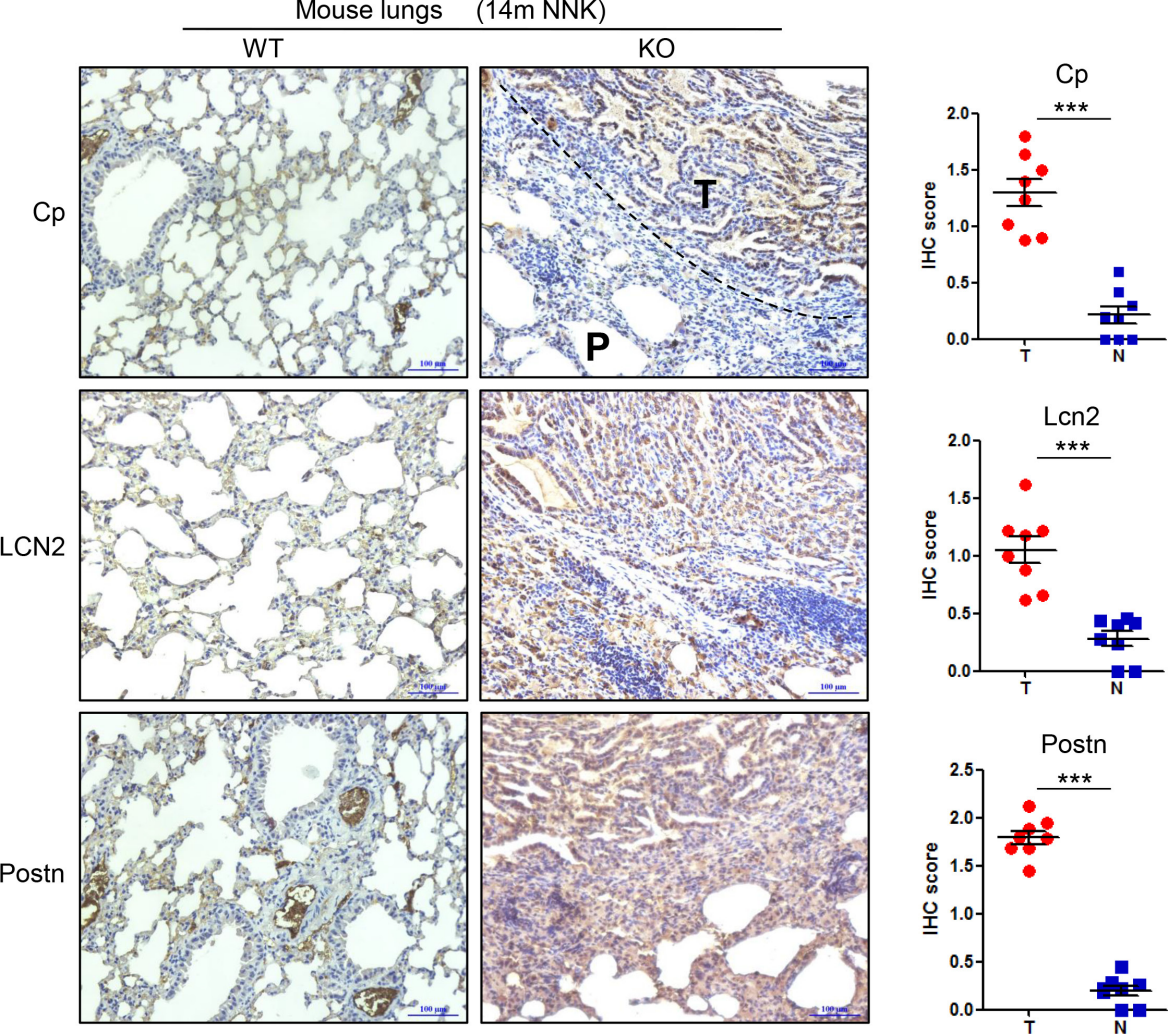
Ceruloplasmin, lipocalin 2 and periostin are upregulated in the lung tumor tissues of Gprc5a-knockout mice compared to wild-type ones Representative images (left) and IHC score (right) of immunohistochemical staining (IHC) of ceruloplasmin (Cp) (T as tumor area, P as paratumor area), lipocalin 2 (LCN2) and periostin (Postn) in the tumor-containing lungs tissues (T) of Gprc5a-ko (KO) mice and the normal lung tissues (N) of wild-type (WT) mice at age of 14 month (14m NNK) (*n* = 8/group). These mice were treated with carcinogen NNK at age of two month.

Next, we asked whether upregulated Cp, LCN2, and Postn, induced by Gprc5a deficiency, are maintained during the process of lung tumorigenesis in Gprc5a-ko mice. By examining the protein levels of Cp, LCN2 and Postn in the lung tissues from wild-type (WT) and Gprc5a-ko mice via immunohistochemical (IHC) staining assay, we found that Cp, LCN2, and Postn protein levels were all significantly increased in tumor-containing tissues (T) from Gprc5a-ko mouse lungs, compared to wild type ones (N) (Figure 3). This suggests that, upregulated Cp, LCN2 and Postn genes are associated with the pathological process of lung tumorigenesis in Gprc5a-ko mice *in vivo*.

### Cp, LCN2 and POSTN are significantly upregulated in NSCLC tissues

**Figure 4:**
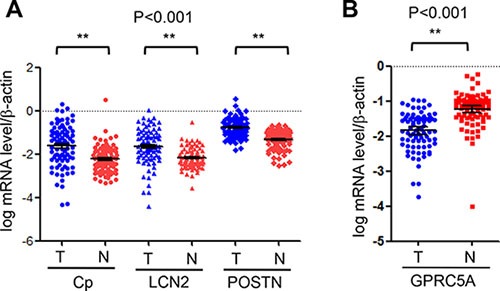
Relative mRNA levels of GPRC5A, ceruloplasmin, lipocalin 2 and periostin in lung tissues of NSCLC Relative mRNA levels of ceruloplasmin (Cp), lipocalin 2 (LCN2) and periostin (POSTN) (**A**) and GPRC5A (**B**) vs β-actin in NSCLC (*n* = 190, or 95 pairs) were measured by Q-PCR and expressed as dot plot as indicated.

To determine if Cp, LCN2, and POSTN are upregulated in human NSCLC, we examined mRNA of these genes in 95 paired NSCLC and adjacent normal lung tissues (Table 1) by Q-PCR analysis. The results of Q-PCR analysis showed that Cp, LCN2, and POSTN were all significantly upregulated in NSCLC tissues (T) compared to adjacent normal lung tissues (N) (Table 2, Figure 4A). In contrast, GPRC5A was significantly downregulated in NSCLC compared to normal lungs (Table 2, Figure 4B). Interestingly, relative expression level of ceruloplasmin is significantly higher in ADC than in SCC, in female than male, whereas relative expression level of periotin is significantly higher in SCC than in ADC, slightly higher in male than in female although no significance (Tables 3 and 5). The difference of ceruloplasmin and periostin in male and female could not be explained by smoking history, since there is no difference in the parameter. In addition, relative mRNA expression levels of ceruloplasmin and lipocalin 2 are not correlated with other parameters, including tumor size, lymph node, TNM stage, histopathological type, and smoking history (Tables 3, 4, 5), suggesting that upregulation of ceruloplasmin and lipocalin 2 is not involved in tumor progression. On contrary, relative expression of periostin is correlated with TNM although is not correlated with other parameters (Table 5), suggesting that upregulation of periostin may be involved in tumor progression in NSCLC.

**Table 2:** Expression of Ceruloplasmin, Lipocalin2, Periostin or Gprc5a mRNA in tumor and adjacent tissues of NSCLC patients

Group	Ceruloplasmin mRNA	*P*-value
Tumor tissue	0.817 ± 0.088	0.000***
Adjacent tissue	0.000 ± 0.057	
**Group**	**Lipocalin2 mRNA**	***P*-value**
Tumor tissue	0.601 ± 0.070	0.000***
Adjacent tissue	0.000 ± 0.045	
**Group**	**Periostin mRNA**	***P*-value**
Tumor tissue	0.569 ± 0.046	0.000***
Adjacent tissue	0.000 ± 0.044	
**Group**	**Gprc5a mRNA**	***P*-value**
Tumor tissue	–0.424 ± 0.013	0.000***
Adjacent tissue	–0.301 ± 0.009	

Data are presented as the mean ± SEM. *P*-value < 0.05 was defined as significant. NSCLC = non-small cell lung cancer.

**Table 3:** Relative expression of ceruloplasmin in NSCLC cancer tissue and its correlation with clinical characteristics of NSCLC patients

Parameter	Ceruloplasmin mRNA	*P*-value
Age, years		
< 60	0.669 ± 0.130	0.144
≥ 60	0.920 ± 0.110	
Gender		
Male	0.680 ± 0.096	0.002**
Female	1.299 ± 0.140	
Pathological type		
ADC	1.109 ± 0.661	0.000***
SCC	0.339 ± 0.142	
T-primary tumor size		
T1 + T2	0.839 ± 0.090	0.428
T3 + T4	0.608 ± 0.254	
N-regional lymph node		
Negative	0.916 ± 0.103	0.126
Positive	0.647 ± 0.145	
TNM stage		
I + II	0.905 ± 0.094	0.082
III + IV	0.571 ± 0.180	
Histopathological type		
Grade 1–2	0.809 ± 0.184	0.951
Grade 3	0.820 ± 0.092	
Smoking history		
Yes	0.785 ± 0.172	0.681
No	0.863 ± 0.095	

Data are presented as the mean ± SEM. *P*-value < 0.05 was defined as significant. NSCLC = non-small cell lung cancer, ADC = adenocarcinoma, SCC = squamous cell carcinoma.

**Table 4:** Relative expression of lipocalin2 in NSCLC cancer tissue and its correlation with clinical characteristics of NSCLC patients

Parameter	Lipocalin2 mRNA	*P*-value
Age, years		
< 60	0.476 ± 0.099	0.160
≥ 60	0.688 ± 0.113	
Gender		
Male	0.583 ± 0.094	0.670
Female	0.664 ± 0.132	
Pathological type		
ADC	0.607 ± 0.092	0.929
SCC	0.592 ± 0.143	
T-primary tumor size		
T1 + T2	0.612 ± 0.079	0.655
T3 + T4	0.492 ± 0.343	
N-regional lymph node		
Negative	0.686 ± 0.101	0.154
Positive	0.455 ± 0.122	
TNM stage		
I + II	0.645 ± 0.091	0.346
III + IV	0.477 ± 0.156	
Histopathological type		
Grade 1–2	0.689 ± 0.148	0.457
Grade 3	0.562 ± 0.092	
Smoking history		
Yes	0.852 ± 0.191	0.110
No	0.511 ± 0.080	

Data are presented as the mean ± SEM. *P*-value < 0.05 was defined as significant. NSCLC = non-small cell lung cancer, ADC = adenocarcinoma, SCC = squamous cell carcinoma.

**Table 5:** Relative expression of periostin in NSCLC cancer tissue and its correlation with clinical characteristics of NSCLC patients

Parameter	Periostin mRNA	P-value
Age, years		
< 60	0.677 ± 0.092	0.137
≥ 60	0.493 ± 0.079	
Gender		
Male	0.622 ± 0.072	0.098
Female	0.381 ± 0.093	
Pathological type		
ADC	0.464 ± 0.069	0.025*
SCC	0.741 ± 0.109	
T-primary tumor size		
T1 + T2	0.535 ± 0.063	0.087
T3 + T4	0.889 ± 0.181	
N-regional lymph node		
Negative	0.531 ± 0.081	0.413
Positive	0.634 ± 0.090	
TNM stage		
I + II	0.495 ± 0.072	0.040*
III + IV	0.776 ± 0.100	
Histopathological type		
Grade 1–2	0.460 ± 0.126	0.236
Grade 3	0.546 ± 0.067	
Smoking history		
Yes	0.668 ± 0.125	0.290
No	0.521 ± 0.070	

Data are presented as the mean ± SEM. *P*-value < 0.05 was defined as significant. NSCLC = non-small cell lung cancer, ADC = adenocarcinoma, SCC = squamous cell carcinoma.

**Figure 5:**
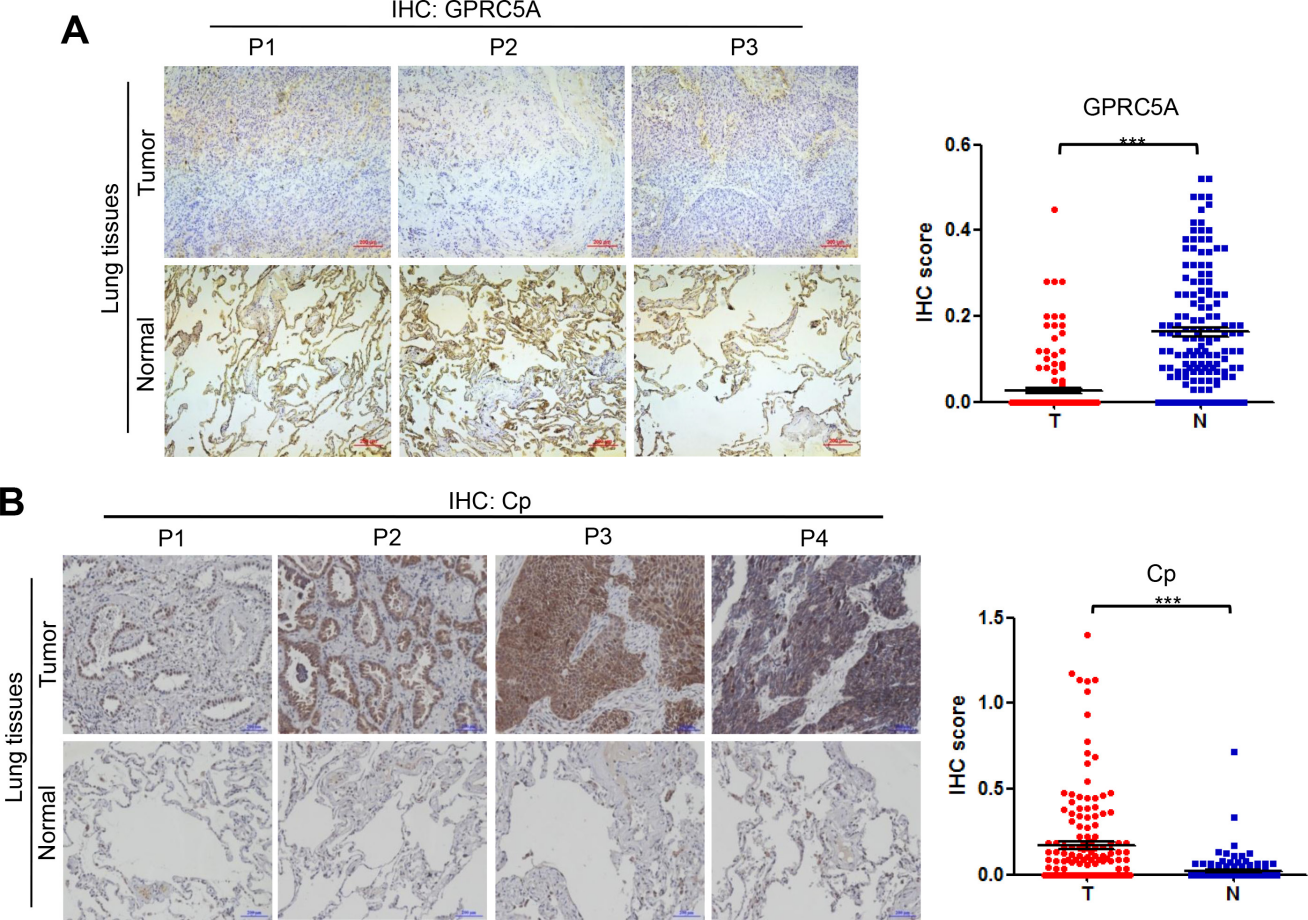
Protein levels of GPRC5A and ceruloplasmin in lung tissues of NSCLC Representative images (left) and IHC score (right), as dot plot, of IHC staining of GPRC5A (**A**) and ceruloplasmin (Cp) (**B**) in NSCLC (*n* = 302, or 151 pairs) were as indicated.

**Figure 6:**
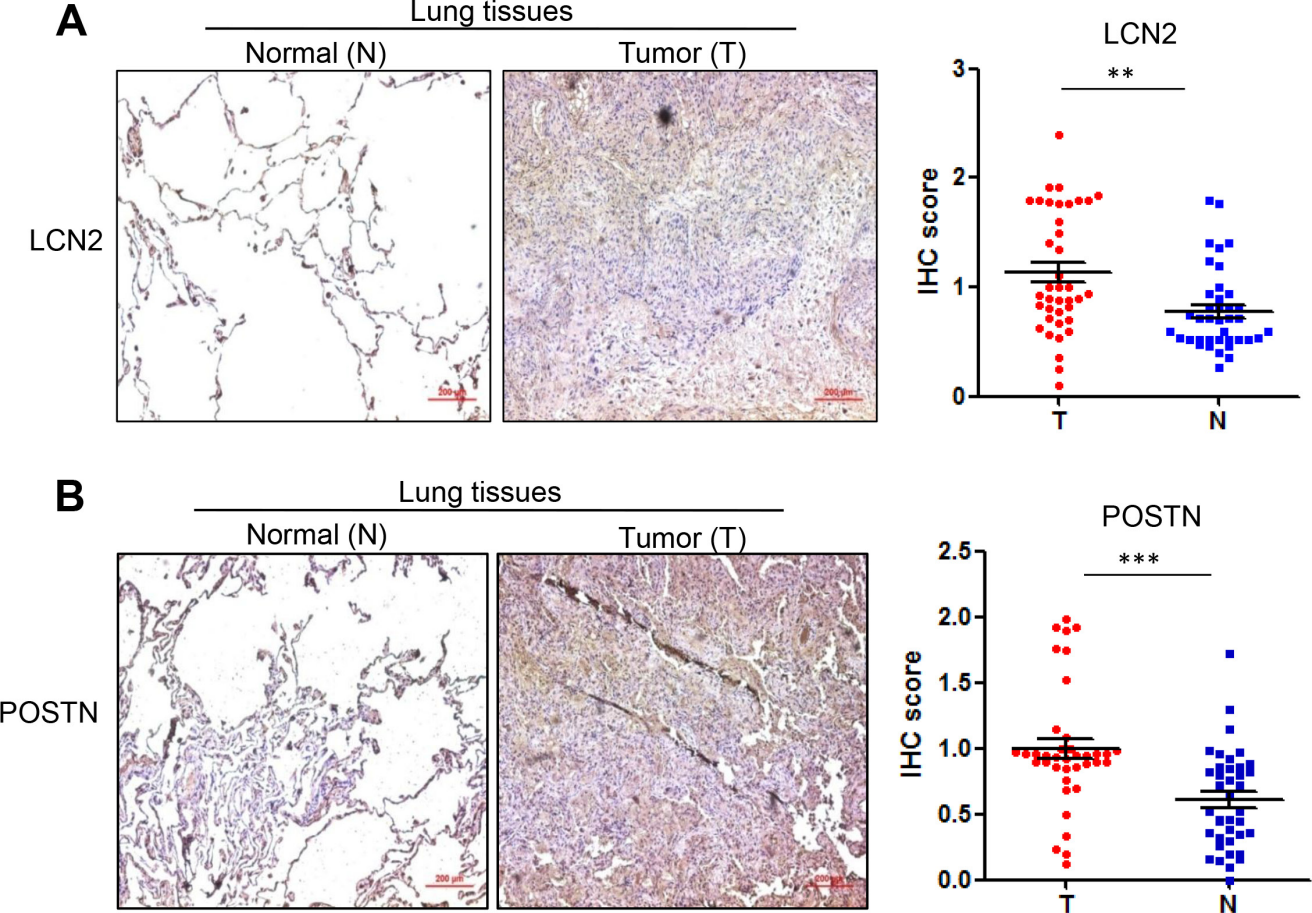
Protein levels of lipocalin2 and periostin in lung tissues of NSCLC Representative images (left) and IHC score (right) of IHC staining of lipocalin 2 (**A**) and periostin (**B**) in NSCLC (*n* = 78 or 39 pairs).

Finally, we measured the protein levels of ceruloplasmin, lipocalin 2 and periostin, as well as GPRC5A, in NSCLC tissues by IHC staining assay. The results of IHC staining showed that GPRC5A level is significantly decreased in NSCLC compared to adjacent normal tissues (Figure 5A), ceruloplamsin (Cp) level is significantly increased in NSCLC tissues (T) compared to adjacent normal tissues (N) (Figure 5B), perioston (POSTN) and lipocalin 2 (LCN2) were significantly increased in NSCLC tissues compared to normal lung tissues (Figure 6A–6B). The protein levels are consistent with mRNA analysis of these genes in NSCLC (Figure 5). Taken together, these results suggest that upregulation of ceruloplasmin, lipocalin 2 and perioston are associated with development of lung cancer, and correlated with repression of GPRC5A in NSCLC.

## DISCUSSION

In this study, we showed that, ceruloplasmin, lipocalin 2 and periostin are upregulated in Gprc5a-ko lung epithelial cells, and importantly, dysregulation of these genes is maintained during lung tumorigenesis of Gprc5a-ko mice. Moreover, ceruloplasmin, lipocalin 2 and periostin are all significantly increased in NSCLC tissues compared to normal lung tissues. These results suggest that, dysregulated ceruloplasmin, lipocalin 2 and periostin may be used as potential biomarkers at early stages of lung cancer development.

It is proposed that, development of solid tumors ordinarily requires five to eight genetic driver gene mutations, and more inactivation of tumor suppressor genes than activation of oncogenes are involved in the process [3]. Previously, we have shown that Gprc5a deficiency lead to spontaneous lung tumor development in mice [5, 9]. Moreover, repression of GPRC5A has been observed in most of NSCLC lung tissues and all of COPD lung tissues [7]. These observations suggest that repression of GPRC5A is involved in initiation of lung tumorigenesis. Thus, the dysregulated genes, ceruloplasmin, lipocalin 2 and periostin, which result from GPRC5A repression, may be used as biomarkers at early stages of lung tumor development.

Ceruloplasmin is the major copper-carrying protein in the blood, with both anti- and pro-oxidant activities. Ceruloplasmin is mainly expressed in the liver. However, lung is another tissue of ceruloplasmin synthesis. It has been reported that ceruloplasmin levels were increased in sera of patients with various acute inflammatory conditions, including injury, malignancy, cardiovascular disease, infection [13–15], and adenocarcinoma lung cancer patients [16]. However, the molecular pathway underlying ceruloplasmin upregulation is unclear. In this study, we show that ceruloplasmin is upregulated via Gprc5a gene deletion in normal mouse lung epithelial cells, and in human NSCLC cells and tissues. Previously, we showed that Gprc5a deficiency confers the susceptibility of Il-6 induction and STAT3 activation, which associates with lung tumor development [6, 9, 17]. Of notion, IL-6 has been implicated in upregulation of ceruloplasmin [12]. Whether IL-6/STAT3 pathway is required for mediating the upregulation of ceruloplasmin in the lung tissues of Gprc5a-ko mice requires further investigation. Interestingly, ceruloplasmin level is significantly higher in lung adenocarcinoma than squamous cell carcinoma, and in female than in male, which could not be explained by smoking history. It is said that ceruloplasmin can be a marker of inflammatory reaction and pain. However, there is no parameter to show the inflammatory status of lungs in these patients. The mechanisms underlying the difference remain for further investigation.

Lipocalin 2 is a member of a larger family of lipocalins. LCN2 gene encodes the protein neutrophil gelatinase-associated lipocalin (NGAL). This protein can bind gelatinase/matrix metalloproteinase-9 (MMP-9) and mediate apoptosis resistance [18]. Lipocalin family proteins share a common tertiary structure that confers the ability to bind and transport a wide variety of lipophilic substances, such as retinoids, fatty acids cholesterol and prostaglandins [19]. Lipocalin 2 has been identified as a stress protein that is released in a variety of other sterile inflammatory conditions such as adipose obesity-related inflammation [20] and cancer. Recently, it has been reported that Lipocalin 2 is associated with radioresistance in oral cancer and lung cancer cells [21], and erlotinib resistance in NSCLC [22]. Functionally, Lipocalin 2 has been suggested in promoting tumorigenesis through enhancing tumor cell survival and proliferation, and metastatic potential [23–25]. In our study, we found that upregulation of Lipocalin 2 can be induced by Gprc5a gene deletion in mouse lung epithelial cells, which is associated with lung tumorigenesis. Interestingly, upregulated Lipocalin 2 is not correlated with any of other parameters including tumor size, lymph node, TNM stage, histopathological type, and smoking history. This supports that Lipocalin 2 may be used as a biomarker at early stages of lung tumor development but not tumor progression.

The periostin protein is a component of the extracellular matrix, which is expressed by fibroblasts. Periostin is expressed by majority of normal adult tissues, including the adrenal glands, lung, thyroid, stomach, colon, vagina, ovary, testis and prostate. Periostin is involved in the maintenance and development of tooth and bone tissue, in addition to cardiac development and healing [26]. Periostin has been observed in a variety of human malignancies [27], including pancreatic, ovarian, colon, lung, breast, gastric, thyroid, and esophageal head and neck carcinomas. Recently, overexpression of periostin has been shown to predict a poor prognosis in NSCLC [27]. It has been shown that the patients with tumor exhibiting high-level periostin expression showed a significantly shorter survival time [27]. Periostin may also contribute to cisplatin resistance in NSCLC cells [28]. Immunohistochemical staining indicated that high levels of periostin were present in the mesenchymal areas, but not in the cancer cells themseleves. Periostin has been shown to promote the proliferation and migration of the human lung ADC cell line (A549) *in vitro* by the EMT pathway (18). Periostin may be induced by transforming growth factor-β (TGF-β) [29]. Periostin is suggested to be required for cancer stem cell maintenance and metastasis (23). In this study, periostin was shown to be induced by Gprc5a gene deletion. Our recent study showed that increased levels of TGFβ, fibrogenic response and EMT-like features as well as neoplasia were induced in the lungs of Gprc5a-ko mice compared to wild-type mice following exposure to silica nanoparticles [30]. In this study, increased periostin is correlated with TNM, suggesting that periostin expression is involved in the process of not only the initiation but also the progression of NSCLC. Interestingly, periostin level is significantly higher in squamous cell carcinoma than in adenocarcinoma, and slightly higher in male than in female although not statistically, which is opposite to ceruloplasmin. This suggests that ceruloplasmin and periostin may be better biomarker for lung ADC and SCC, respectively. The underlying mechanism requires further investigation.

Ceruloplasmin, lipocalin 2 and periostin are detectable in serum, which is applicable as biomarkers for early diagnosis. Thus, future work will be focus on analysis of ceruloplasmin, lipocalin 2 and periostin in the serum samples of NSCLC patients. Taken together, our study suggests that ceruloplasmin, lipocalin 2 and periostin are potential candidate biomarkers at early stages for lung cancer.

## MATERIALS AND METHODS

### Patients

Lung specimens from cancer tissues and paired paratumor tissues (with 1–2 cm distance from tumor edge) and normal tissues (with > 5 cm distance from tumor edge) from 95 NSCLC patients (Table 1), who underwent pulmonary resection surgery, were included in this study. The samples were obtained by the Department of General Surgery of Shanghai Chest Hospital (Shanghai, China) between May 2013 and November 2014. All diagnoses were based on pathological evidence. Patients were grouped according to the size of the primary tumor (T), nodal involvement (N) and distant metastasis (M) to TNM stages I–IV according to the World Health Organization criteria for the TNM system and staged appropriately. Patients did not receive chemo-, radio- or immunotherapy prior to surgery. The tissues were snap frozen and stored at –80°C until use. This study was authorized by the principle committee of Shanghai Chest Hospital. Written informed consent was obtained from the patients.

### Bioinformatic analysis of mRNA expression using oncomine cancer gene microarray database

The microarray data showed significant overexpression of 20 genes. In order to investigate these genes expression, mRNA expression of lung cancer samples and normal samples were obtained from Oncomine^TM^. The database provided as Log2 median RNA expression values and the *P* value is 1E-4 FOLD CHANGE is 2 GENE RANK is 10%.

### Q-PCR assay

Total RNAs were extracted from tissues with TRIzol reagent (Invitrogen). The levels of mRNA were quantified by quantitative reverse transcription PCR (qRT-PCR) using SYBR Green (Takara), with beta-actin as internal normalized references, The qRT-PCR results were analyzed and shown mRNA levels of the CT (cycle threshold) values, which were then converted as fold change.

The primer sequences for qRT-PCR are:

Cp-F: TATCCGTGGGAAGCATGTTAGA, Cp-R: TGT GTACTCACGATAAACCAGC; Postn-F: CTCATAGTCG TATCAGGGGTCG, Postn-R: ACACAGTCGTTTTCTGT CCAC; LCN2-F: GAAGTGTGACTACTGGATCAGGA, LCN2-R: ACCACTCGGACGAGGTAACT; β-ACTIN-F:iCACCACGGCCGAGCGGGAAATCGT, β-ACTIN-R: CCTCAGGGCAGCGGAACCGCTCAT.

### Immunoblot

Cells were lysed with RIPA lysis buffer (Cell Signaling Technology). Cell lysates were separated by SDS/PAGE in a 10% acrylamide gel and transferred onto nitrocellulose membrane for immunoblot as described previously [31, 32]. Antibodies against ceruloplasmin (Santa Cruz, sc-20957), lipocalin 2 (abcam, ab63929), periostin (abcam, ab14041), GPRC5A (Santa Cruz, sc-98885), and β-actin (Cell Signaling Technology, 5125) are as indicated.

### Immunohistochemistry (IHC)

The expression of CERULOPLASMIN in the human lung tumor tissue chip of the cohort of 302 tissues, including 151 available para-tumor tissues, was examined by Immunohistochemistry (IHC). Formalin-fixed, paraffin-embedded archived tissues were cut into 5-μm section. Every slide contained about 74 tissue points and there were four slides. Then the sections were de-waxed in xylene and rehydreated with ethanol arranged a graded concentration. 0.3% hydrogen peroxide blocked the endogenic peroxidase, after then, the antigens were retrieved in All-purpose Powerful Antigen Retrieval Solution (Beyotime,P0088) for 20 minutes in boil water and cooled naturally to room temperature. The sections were incubated overnight as 4°C with rabbit polyclonal antibody against human CERULOPLASMIN (Santa Cruz Biotech, sc-20957, 1:100), LIPOCALIN 2 (abcam, ab63929, 1:100) and PERIOSTIN (abcam, ab14041, 1:100). Then the sections were detected with the Detect System/Mo&Rb Kit (GTVison, GK500705). Negative controls were employed in which the primary antibody was rabbit normal IgG.

### IHC staining evaluation

To estimate the expression of CERULOPLASMIN, LIPOCALIN 2 AND PERIOSTIN in tumor or para-tumor, we improved the score system as described previously. The expression of these three genes was scored with intensity of staining and the percentage of the cells of interest staining. We ranked the intensity of staining into 4 categories: 0 (–), 1 (+), 2 (++), and 3(+++). The (–), (+), (++), (+++) were defined as no staining, weak staining, moderate staining and intense staining, respectively. Then we evaluated the percentage of positive staining and grouped them into 4 categories: 0 (0%), 1 (1%–29%), 2 (30%–69%), and 3 (≥ 70%) (Supplementary Figure 1). The total score of one tissue point was obtained by multiplying the intensity and percentage score. Each section was independently scored by two pathologists. The result was analyzed with the non-parametric alternative to ANOVA. Here we used the Kruskal-Wallis H to test for difference between four independent groups, if there was significantly difference, then we used the Mann-Whitney U to test for difference between every two groups. The final statistical graph was showed in GraphPad Prism 5.

### Statistical analysis

The data analysis was performed using SPSS (Statistic Package for Social Sciences) 23.0. Values are presented as the mean ± SEM. The significant differences between tumor tissues and adjacent tissues were analyzed by the paired samples *t-test*. The correlation between gene expression and the clinicopathological characteristics of the NSCLC patients was calculated based on independent samples *t-test*. A value of *P <* 0.05 was considered statistically significant.

## SUPPLEMENTARY TABLE AND FIGURE


