# Supplementary material for: Gprc5a-knockout mouse lung epithelial cells predicts ceruloplasmin, lipocalin 2 and periostin as potential biomarkers at early stages of lung tumorigenesis
Source: Oncotarget. 2017 Jan 10;8(8):13532–44. doi: 10.18632/oncotarget.14589 (PMC5355118; doi:10.18632/oncotarget.14589)
Supplement: Supplementary file 1 [file oncotarget-08-13532-s001.pdf]

Gprc5a-knockout mouse lung epithelial cells predicts ceruloplasmin, lipocalin 2 and periostin as potential biomarkers during early stage of lung tumorigenesis

Supplementary Materials

Supplementalry Table 1: Upregulated genes in Gprc5a-ko MTEC vs wild-type MTEC

Probe Set ID	Gene Symbol	Gene Description	Fold change	mRNA Accession
6769535	Timp3	tissue inhibitor of metalloproteinase 3	91.08420554	NM_011595
6833382	Krt18	keratin 18	88.13340261	NM_010664
7010835	Gria3	glutamate receptor, ionotropic, AMPA3 (alpha 3)	77.72511818	NM_016886
**6885873**	**Lcn2**	**lipocalin 2**	**51.97712747**	**NM_008491**
6824932	Gjb2	gap junction protein, beta 2	48.50592237	NM_008125
7018847	Itm2a	integral membrane protein 2A	41.55810600	NM_008409
6783847	Hoxb2	homeobox B2	40.14487733	NM_134032
6901680	Ddit4l	DNA-damage-inducible transcript 4-like	35.61312178	NM_030143
6772802	Enpp1	ectonucleotide pyrophosphatase/phosphodiesterase 1	29.45427220	NM_008813
7010390	Slc6a14	solute carrier family 6 (neurotransmitter transporter), member 14	28.42524739	NM_020049
**6896032**	**Cp**	**ceruloplasmin**	**27.49577412**	**NM_001042611**
6763208	Mr1	major histocompatibility complex, class I-related	26.69143564	NM_008209
6875214	Plxdc2	plexin domain containing 2	26.01372331	NM_026162
6859285	Dsg2	desmoglein 2	24.79841486	NM_007883
6849109	Zfp760	zinc finger protein 760	21.17850724	NM_001008501
6950582	Mgst1	microsomal glutathione S-transferase 1	20.42349690	NM_019946
6823887	Sncg	synuclein, gamma	19.67014926	NM_011430
6762094	Cd55	CD55 antigen	19.34169064	NM_010016
6882256	2310046K01Rik	RIKEN cDNA 2310046K01 gene	18.65359766	NM_027172
**6897557**	**Postn**	**periostin, osteoblast specific factor**	**18.14455790**	**NM_015784**

MTEC = mouse tracheal epithelial cells.

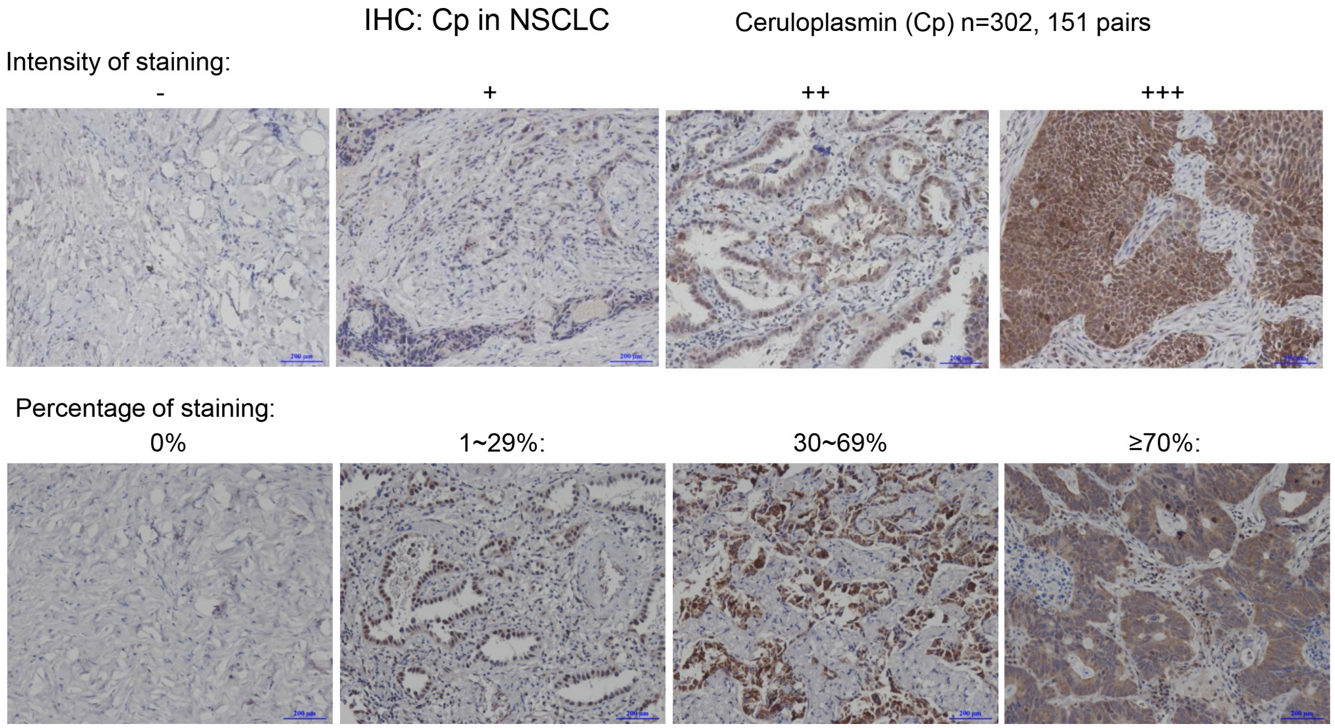

Supplementary Figure 1: The examples of IHC Cp staining. The intensity of IHC staining were rated into 4 grades: 0 (–), 1 (+), 2 (++), and 3 (+++) as indicated.
